# A Conference (Missingness in Action) to Address Missingness in Data and AI in Health Care: Qualitative Thematic Analysis

**DOI:** 10.2196/49314

**Published:** 2023-11-23

**Authors:** Christian Rose, Rachel Barber, Carl Preiksaitis, Ireh Kim, Nikesh Mishra, Kristen Kayser, Italo Brown, Michael Gisondi

**Affiliations:** 1 Department of Emergency Medicine Stanford University School of Medicine Palo Alto, CA United States; 2 Stanford University Palo Alto, CA United States

**Keywords:** machine learning, artificial intelligence, health care data, data quality, thematic analysis, AI, implementation, digital conference, data quality, trust, privacy, predictive model, health care community

## Abstract

**Background:**

Missingness in health care data poses significant challenges in the development and implementation of artificial intelligence (AI) and machine learning solutions. Identifying and addressing these challenges is critical to ensuring the continued growth and accuracy of these models as well as their equitable and effective use in health care settings.

**Objective:**

This study aims to explore the challenges, opportunities, and potential solutions related to missingness in health care data for AI applications through the conduct of a digital conference and thematic analysis of conference proceedings.

**Methods:**

A digital conference was held in September 2022, attracting 861 registered participants, with 164 (19%) attending the live event. The conference featured presentations and panel discussions by experts in AI, machine learning, and health care. Transcripts of the event were analyzed using the stepwise framework of Braun and Clark to identify key themes related to missingness in health care data.

**Results:**

Three principal themes—data quality and bias, human input in model development, and trust and privacy—emerged from the analysis. Topics included the accuracy of predictive models, lack of inclusion of underrepresented communities, partnership with physicians and other populations, challenges with sensitive health care data, and fostering trust with patients and the health care community.

**Conclusions:**

Addressing the challenges of data quality, human input, and trust is vital when devising and using machine learning algorithms in health care. Recommendations include expanding data collection efforts to reduce gaps and biases, involving medical professionals in the development and implementation of AI models, and developing clear ethical guidelines to safeguard patient privacy. Further research and ongoing discussions are needed to ensure these conclusions remain relevant as health care and AI continue to evolve.

## Introduction

Machine learning offers great promise to rapidly analyze data and provide decision support that can bolster efforts to mitigate health disparities and achieve high-quality health outcomes. Effective machine learning requires robust, balanced, and comprehensive data upon which to build accurate models. Given that the success of machine learning relies on the availability of data inputs, the growth in machine learning has reinforced additional rapid growth in health care data and records over the past several years [1-4].

This “missingness” in data is common in medicine and medical records [5,6]. Missingness in health care data refers to the absence or incompleteness of health-related information for certain individuals or variables, which can occur due to various reasons such as nonresponse, loss to follow-up, or systematic errors in data collection or recording. For example, machine learning applications attempting to address maternal mortality are severely limited by inconsistent data collection at a state level and incomplete statistics [7]. Even the largest clinical data sets contain information on only a small fraction of the population and represent those communities that have historically benefitted from the greatest access to health care [8]. The resulting imbalances and lack of representation of certain populations may be the largest barrier to the generalizability of technologies that rely on health care data [9,10].

Understanding how to control for missing information—which includes identifying and filling gaps and incorporating data we do not yet measure—is critical to the successful, equitable implementation of machine learning in health care. Without improved, accurate, and comprehensive data, even the best models will face limitations in their predictive abilities. While some techniques for managing and inferring missing data exist, their efficacy is limited for data that are missing “not at random,” that is, missing not simply due to chance but rather due to the lack of representation or structural biases in the delivery of medical care. Taken further, this means that missing data may be more likely for underserved or marginalized populations, which thereby significantly limits the use of models and the associated techniques to handle missing data and, in turn, hampers the clinical applicability of the resulting tools for the patient populations who may need them most [11,12].

Furthermore, while we have developed methods to infer gaps within existing data sets, little is known about how best to determine the comprehensiveness of a data set or how to acquire information that may be missing [13]. Community-based approaches, such as censusing, are possible but must be carefully considered to avoid unintended side effects. Even selecting what to measure is a product of societal and cultural values, and useful data for one population may lead to bias in another [14]. A community-based participatory approach to building and collecting health care data sets is needed, but the path toward that goal is not clear.

In September 2022, we hosted *Missingness in Action: A Stanford Conference on the Absence of Data and the Future of Artificial Intelligence (AI) in Healthcare*. Our primary aim for the conference was to educate participants about the problem of missingness in health care data and to gain insights into how to address this problem. In this paper, we report (1) the methods used to execute this digital conference, (2) conference attendee engagement and analytics, (3) qualitative analysis of conference discussions, and (4) proposed strategies for addressing missingness in health care data.

## Methods

### Study Design, Setting, and Population

Our research methodology was driven by a “conference as research” approach, which has been used previously by members of the research team [15,16]. The motivation for this approach and the thematic analysis of conference data was rooted in the unique advantages offered by this setting. Unlike a review of published work or other traditional forms of qualitative inquiry, the conference facilitated real-time multifaceted dialogue among diverse experts, capturing rich, current, and interactive perspectives on the impact of missing data on AI in medicine. Moreover, the consensus building that unfolded during the conference, coupled with public accessibility, provided an unparalleled depth to the discourse and allowed for broad stakeholder engagement.

Using a constructivist paradigm, we conducted a thematic analysis of the 4-hour *Missingness in Action* conference transcript [17]. Our study aim was to identify strategies and practices necessary to account for missing data and its impact on AI in medicine. A transcript of the conference proceedings served as the data for the study, and the results of our thematic analysis represent the primary study outcomes.

The conference was called *Missingness in Action: A Stanford Conference on the Absence of Data and the Future of AI in Healthcare*, and it was sponsored by the Department of Emergency Medicine at Stanford School of Medicine and the Stanford Human-Centered Artificial Intelligence (HAI) program. The conference took place on September 22, 2022, and we performed data analysis between November 2022 and February 2023. The conference speakers agreed to be video recorded during the event, and the video is now in the public domain [17]. Therefore, we did not seek further consent for the purpose of data analysis. The complete video recording of the conference is publicly available [17]. This represents the raw data used in this qualitative study.

### Ethical Considerations

The Stanford School of Medicine institutional review board deemed this study exempt (IRB# 64930).

### Study Intervention

Deliberate design and successful execution of the conference were integral to the collection of meaningful data for this study. We convened a planning committee that met weekly for 6 months prior to the conference comprising 3 faculty members from the Stanford University Department of Emergency Medicine (CR, IB, and MG) and 2 Stanford undergraduate students (RB and NM). The planning committee was responsible for obtaining a grant to fund the conference from Stanford HAI (CR, IB, and MG), selecting and recruiting speakers, determining the date of the conference, and managing the web-based conference platform in real time [18]. This committee organized a conference agenda consisting of several panel discussions and mini–keynote lectures on broad topics related to the goals of the conference and the objectives of this study. The committee used a diversity and inclusion lens to recruit expert participants across many relevant fields from diverse geographic, cultural, and academic backgrounds. The conference date was chosen based on speaker availability and to provide the time necessary for planning. The conference was hosted digitally and without a fee so that it could be available to a general audience. It was open access to the public to ensure broad engagement from interested individuals.

The conference committee developed a conference website using Squarespace (Squarespace Inc) that served as the registration portal, conference platform, and information source for social media and digital marketing [17]. The marketing of the conference was conducted through Twitter (Twitter Inc; subsequently rebranded as X) and Stanford Medicine newsletters and email distribution lists. Twitter was also used for same-day conference backchanneling [19]. Participants were asked to share information about the conference with potential attendees through direct outreach and on their social media accounts, and we provided marketing materials for this purpose. Hopin (Hopin Ltd) was chosen as the digital conference platform as it met the needs for audience polling and interaction, question and answers, and having multiple speakers on the screen at once. RB managed the digital platform on the conference day including speaker logins, backstage preparations, and monitoring live feeds. Additional planning committee members oversaw the audience chat for questions, took notes of the proceedings, and shared highlights on social media in real time.

### Participant Sampling and Data Collection

Purposeful and snowball sampling was used to identify the 13 expert speakers for the conference; these expert speakers represent the study participants [20-22]. Conference topics and agenda were based on the learning objectives, the aims of this study, and the digital format (type and length of presentations, topics, etc). Panelists and speakers were then identified based on their achievements and reputations gleaned from publications, internet searches, and word-of-mouth recommendations. The conference was digitally recorded, and YouTube (Alphabet Inc) was used to generate a transcript of the presentations and panels, which serves as the study data.

### Data Analysis

We performed a thematic analysis of the conference transcript to identify strategies and practices related to missing data and AI use in health care [22,23]. We followed Braun and Clark’s [24] methodology for thematic analysis, which includes 6 steps such as familiarization with the data, generation of codes, combining codes into themes, reviewing themes, determining the significance of themes, and reporting findings. This approach was aligned with our constructivist orientation and allowed flexibility in our data analysis while maintaining transparency and rigor. The team met initially to agree on the coding approach and rules. We inductively coded the transcript for the presence of strategies and practices discussed by the participants, but not their frequency. We analyzed the data to the level of sentences and grouped these into loose concepts or constructs to generate codes and their meanings [25]. Two investigators (RB and NM) coded the data, and they met frequently to discuss new codes and resolve any disagreements. The final codebook represented codes agreed upon by both parties. The larger team then met again to perform a thematic analysis of the codes using a consensus approach [22-24]. Themes were named and defined. We used descriptive statistics to analyze conference participation, engagement, and relevant measures of impact.

### Reflexivity

The experiences and opinions of our investigator team may have biased the data analysis in this constructivist paradigm. We explicitly acknowledged these biases during the coding process and at team meetings. Four of the study authors (CR, IB, MG, and CP) are emergency medicine faculty members at a medical school. The lead investigator (CR) has advanced training in medical informatics. Another team member is an expert in social determinants of health (IB), and the senior author (MG) is an experienced medical education and qualitative researcher. CP is a medical education researcher with formal training in qualitative methods. MG and RB have conference planning experience and qualitative research experience. RB oversaw each methodological step in the data analysis, ensuring consistency and compliance with the a priori coding approach used by other novice investigators (NM), as well as maintaining a research diary of processes and team discussions. The study team had content knowledge about missing data and AI in health care that ranged from novice (IB, MG, RB, NM, and CP) to expert (CR). They reflected on their experiences throughout the conference planning process and during each meeting in which data were discussed.

## Results

### Conference Participation, Engagement, and Analytics

#### Participants

We recruited 20 participants to moderate discussions or deliver presentations during the day of the conference. Individual participants were organized into panels based on overarching questions that guided the discussion for the day (Table 1). The participants who delivered an individual presentation set the background for the panel discussion that followed (Table 1).

**Table 1:** Summary of conference panels and presentations.

Presentation title and speaker		Role	Affiliations
**Opening remarks**			
	Christian Rose	Physician and informaticist	Stanford University
**Your data are missing pieces** **—** **presentation**			
	Ziad Obermeyer	Physician and research scientist	UC Berkeley
**Your data are missing pieces** **—** **panel**			
	Latrice Landry	Research scientist	Dana Farber Cancer Institute
	Pranav Rajpurkar	Research scientist	Harvard University
	Maya Mathur	Statistician and research scientist	Stanford University
	David Kim (Moderator)	Physician and research scientist	Stanford University
**Finding the missing pieces** **—** **panel**			
	Mitch Lunn	Physician and research scientist	Stanford University
	Sharath Guntuku	Research scientist	University of Pennsylvania
	Andrew Marshall	Physician and informaticist	Brigham and Women’s Hospital
	Marinka Zitnik	Research scientist	Harvard University
	Maria Raven	Physician and research scientist	UCSF Medical Center
	Italo Brown (Moderator)	Physician and policy researcher	Stanford University
**Completing the puzzle** **—** **presentation**			
	Andrew Eland	Research scientist	Diagonal Works
**Completing the puzzle** **—** **panel**			
	Krystal Tsosie	Research scientist	Arizona State University
	David Janka	Physician and design expert	Stanford University
	Alcir Santos Neto	Policy researcher	Economist Impact
	Negar Rostamzadeh	Research scientist	Google Research
	Michael Gisondi (Moderator)	Physician and research scientist	Stanford University
**Seeing the whole image**			
	David Magnus	Ethicist	Stanford University

#### Same-Day Engagement

A total of 861 individuals registered for the conference, and 164 (19%) viewed the meeting while it was live. Registration numbers began to climb in early September and peaked on September 17, 2023, with 573 registrations in a single day. The peak attendance at one given time during the conference was 112.

#### Asynchronous Engagement

Once complete, a recording of the conference was hosted on its website. We examined website analytics to measure asynchronous engagement. The conference website had a total of 1725 visits.

### Thematic Analysis

**Figure 1:**
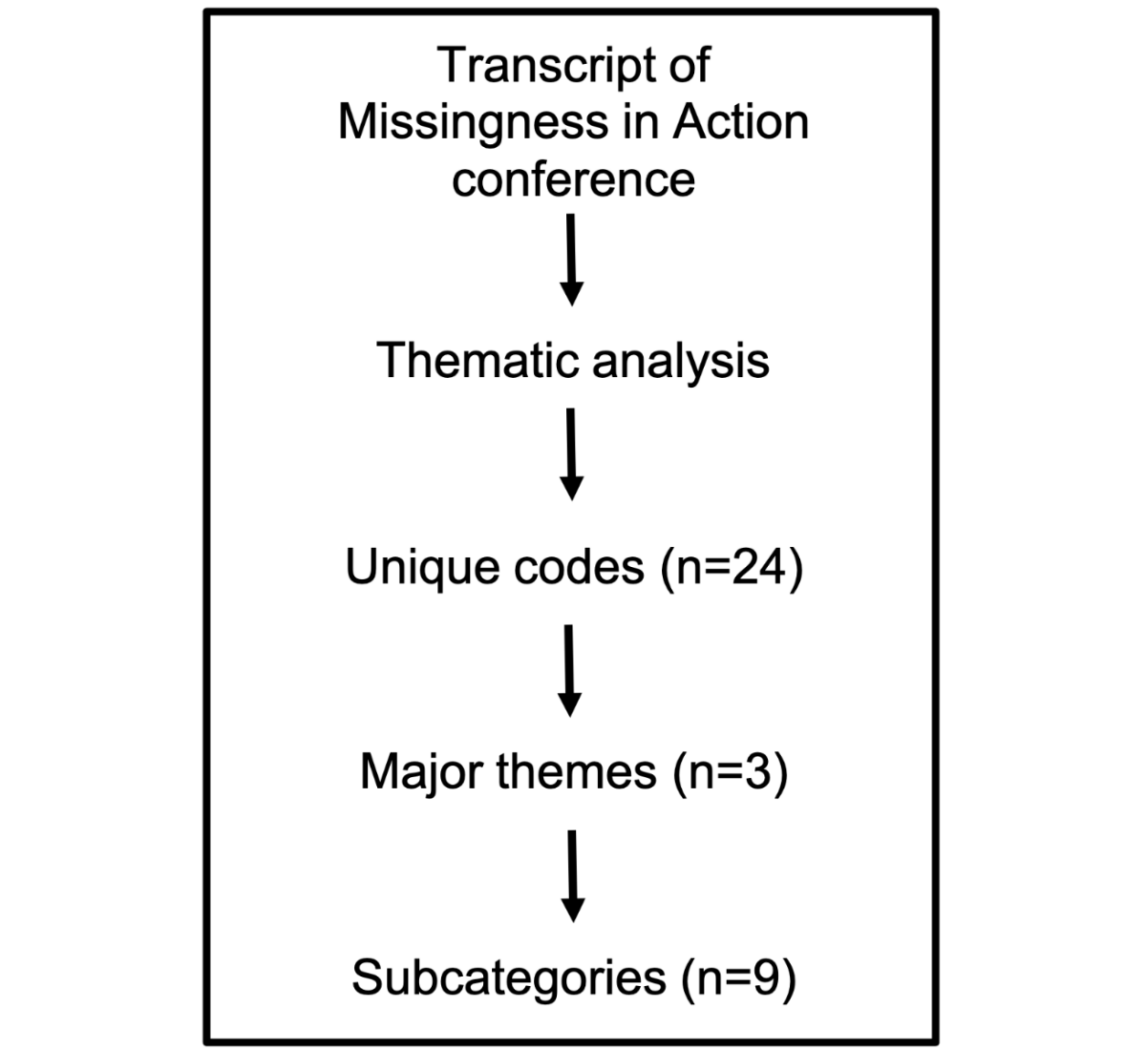
Data analysis overview.

The digital recordings of the 7 individual presentations resulted in 96 pages of transcribed text. Our thematic analysis generated 24 total codes, which were condensed further into themes with subcategories (Figure 1). The 3 overarching themes identified from the complete conference transcript were *data quality and bias*, *human input in model development and deployment*, and *trust and privacy* (Table 2).

**Table 2:** Three themes and 9 subcategories were identified through an analysis of the conference transcript.

Theme and subcategories		Representative quotes
**Data quality and bias**		
	Unrepresentative data affects the generalizability of models to a broader population.	“Those communities who have historically benefited from access to health care may heavily bias results and limit the generalizability of data.”
	Historically, underrepresented communities have been exploited, and trust needs to be rebuilt by allowing participants to be “co-owners” of research projects.	“Making sure that community members and participants see themselves as co-owners of a particular research project shows that you are actually committed to involving communities that have been underserved and underrepresented in the past.”
	Social determinants of health have a significant impact on outcomes; we can only obtain data about these determinants by specifically requesting and including it in our treatment.	“It’s alarming to see the difficulties of data collection and sometimes it doesn’t represent the situations that patients are going through with different social determinants of health. Sometimes, it is critical for us to include this big picture in the conversation and be intentional about asking for this information.”
**Human input in model development and deployment**		
	Machine learning can complement, but not replace, physicians; the supremacy of presence ensures that doctors have more information than an algorithm.	“People feel like they're going to be replaced by the machine...in practice though...medicine is transformative and dynamic and so the algorithm is something that can be trained on sort of an existing evidence-base...but the human will always have more knowledge.”
	In the presence of inevitable missing data, there are 2 ways to proceed—one may have sufficient information to make a probabilistic decision, but if not, one must rely on personal medical judgment.	“Okay, sure we don’t have the literal test result in untested patients, but we do have longitudinal electronic records that let us see, okay, well what happens to this patient, for example, over the next 30 days?”
	The cultural differences between consumer technology and health care lie within the need to contextualize and include medical professionals before deploying.	“Most of the technology we use at the moment has been built from the perspective of consumer technology dealing with data about an individual that's on your phone and Health care is quite different because you have real benefits to society from working on data analysis and you know the data itself it's really difficult to say that any one piece of healthcare data represents solely one individual.”
**Trust and privacy**		
	One can improve the predictive utility of machine learning models in health care by incorporating novel sources of personal data as well as improving the affordability and accessibility of care; however, requesting more from patients requires balancing trust and privacy with accessibility.	“We tried to see if Facebook posts alone can forecast if a person will be depressed, say six months before their official diagnosis or three months before their official diagnosis. And surprisingly, the machine learning model that is trained just on language from patients' social media posts does about, I think, 0.75 AUC.”
	Trust is important to keep data sustainable; this trust is garnered and maintained through community engagement and academic accountability.	“Importantly, it’s making sure that community members and participants see themselves as co-owners of a particular research project and that they are as important as the academic researchers…show that you are actually committed to involving communities that have really been underserved and underrepresented in the past.”
	The sensitivity of health care data necessitates different approaches to data inference; sometimes, introducing noise into the data can keep patient information private and reduce researcher apprehension about data exposure, but simultaneously can make data more difficult to interpret.	“Adding noise to data deliberately to mask the effects of any one individual on values computed from that data set.”

**Themes Identified**

#### Data Quality and Bias

Missingness in health care data stems from the data collection process itself, in which an underrepresentation of historically marginalized or underserved communities and key health factors leads to poor data quality and bias. Underrepresentative data weaken the generalizability of models to a broader population, limiting the application of such data to AI for practice. It is important to identify upstream sources of bias and mitigate their impacts by using inclusive data collection strategies and “debiasing” data through algorithms.

Historically marginalized communities often experience mistrust and reluctance toward research engagement due to past exploitation. This can not only result in underrepresentation but can also lead to danger in medical circumstances, exemplified by a speaker’s statement that:

In clinical trials, there may be missing data on the safety and efficacy of a biomarker in a certain population due to underrepresentation in basic science research.

Underrepresented groups extend beyond racial and ethnic minority groups, including certain socioeconomic or gender groups, or individuals with disabilities. To encourage research engagement in historically marginalized communities, trust must be rebuilt by “allowing participants to be co-owners of research projects” and fully involving them in the research process.

Data quality is also impaired by failing to take into account key health factors that impact patient outcomes. For instance, social determinants of health are often overlooked when building models or treating patients, leading to treatments that are unrealistic or unsuitable for that specific patient. “Patients’ experiences outside of the health care system can be a black box for physicians,” and it is important for physicians and researchers to ask for and incorporate this information into AI models and treatment plans. “If you don’t ask,” 1 speaker noted:

You don’t have the information, and that’s one of the problems in using healthcare data alone- is that as physicians we are actually not very good at asking people...so the problem that creates when we don’t ask is that we don’t have data...we don’t have good data.

It was emphasized that “We need to ask a lot more, we need to have more systematic ways to gather these data on people in the healthcare setting.”

#### Human Input in Model Development and Deployment

Throughout the discussions, numerous speakers and panelists emphasized the critical role of human expertise and professional judgment in the clinical decision-making process. One panelist poignantly remarked, “there is simply too large of a differential between the information that a physician can access and the information that any given model might be able to access.” This vast difference in the amount and dimensionality of data available to human practitioners ensures that physicians will typically make more comprehensive clinical decisions than an algorithm. Reinforcing this point, 1 speaker added:

The physician is always going to see things that we don’t (measure), and that...is going to be useful for the physician’s decision making.

However, as the digitization of clinical observations expands (eg, continuous patient monitoring mechanisms and computer vision during video visits), the gap between automated clinical decision tools and physicians’ opinions may narrow. Consequently, this could lead to improved health outcomes for patients through the application of machine learning, ultimately resulting in enhanced health care outcomes.

In health care settings, human participants not only have access to a greater amount of information, but they also rely on their expertise to determine the best course of action in the face of missing data. For instance, even when algorithms exist for specific diseases, health care professionals must decide whether to obtain the necessary data or make a decision without it. This choice depends on medical judgment, the potential severity of the outcome without the data, and the practical realities of the health care system—an aspect sometimes referred to as medical gestalt.

Conference participants also addressed the challenges of integrating existing technological solutions into medical settings. These difficulties stem, in part, from the fact that many of these technologies were initially developed for other purposes, such as the consumer technology industry. As a result, they may not fully align with the unique requirements and constraints of medical settings, where motivations and incentives, both financial and otherwise, differ significantly. Medical technologies must comply with strict regulations such as the Health Insurance Portability and Accountability Act (HIPAA), which imposes severe financial and legal penalties for violations, irrespective of intent. In contrast, numerous consumer technologies depend on widely accessible, user-provided data for optimal functionality.

#### Trust and Privacy

A central theme that recurred in many of the conference panels and presentations was the importance of trust and privacy when considering the use of data and AI in health care. Stemming from this theme were 2 important aspects: individual privacy and community trust. The first aspect concerns the privacy of patients whose data are being implemented in machine learning models. The second aspect focuses on a wider scope, examining the effect that these models will have on communities, particularly communities that are historically underrepresented.

When examining privacy on an individual level, several speakers discussed concerns surrounding whether an individual’s data might be discerned from data sets, especially when these data sets are smaller. One proposed solution is adding noise to data sets. According to 1 speaker, “adding noise to data deliberately to mask the effects of any one individual on values computed from that data set.” While this would help minimize the potential of any individuals being singled out in data sets, it introduced a new concern—accuracy. The participant explained:

The biggest problem it [adding noise to a data set] sets up this concept of privacy versus accuracy. The more noise you add to a system, the more you protect an individual’s privacy.

There is no consensus as to what the best solution is for maximizing both privacy and accuracy, and this is important to examine in future research.

Examining trust at a community-wide level, it is important to address the relationships between researchers and the communities with which they interact. Many speakers throughout the *Missingness in Action* conference spoke about this relationship. One participant said:

Importantly, it’s making sure that community members and participants see themselves as co-owners of a particular research project and that they are as important as the academic researchers...show that you are actually committed to involving communities that have really been underserved and underrepresented in the past.

Echoing these thoughts, another speaker stressed the importance of participatory research:

Making sure that there’s a way to involve research participants at every step in the research process. So that’s not academic medicine deciding the best research questions to ask, that’s actually the research participants figuring that out.

In other words, to ensure that data sets are truly representative of the population, it is important to include data from previously underrepresented communities and make them a part of the research process. Through fostering a relationship between researchers and the community, a foundation of trust can be built.

## Discussion

### Principal Findings

Missingness threatens to undermine the successful implementation and use of AI and machine learning in health care. Our analysis of the conference proceedings uncovered challenges and potential solutions for addressing missingness in health care data and the impact on AI applications. These included the accuracy of predictive models, lack of inclusion of underrepresented communities, partnership with physicians and other populations, challenges with sensitive health care data, and fostering trust with patients and the health care community. We identified 3 principal themes: *data quality and bias*, *human input in model development*, and *trust and privacy*. Accounting for these themes is vital when devising and using machine learning algorithms in health care. The identified challenges align with the existing literature regarding AI in medicine [26,27], although legal and regulatory concerns were not as large a focus in our study. By acknowledging the challenges of data quality, human input, and trust, we can create systems that are equitable, accurate, and advantageous for all patients.

Data quality and bias are critical issues in developing AI models for health care applications. Applying models at scale requires both representative and comprehensive data. Collecting a broader and fuller complement of data will require new data collection strategies. Community-based participatory approaches are one potential mechanism to gather data from historically underrepresented populations; however, these partnerships may require rebuilding trust with populations that have traditionally been marginalized by the health care system. Many researchers are still working to better understand social predictors of health outcomes, and a robust understanding of structural biases in health care delivery is essential to develop accurate and effective AI models.

The degree of human involvement in the development and deployment of AI models is an ongoing area of debate in the AI community. Potential applications of AI are rapidly accelerating, especially with the recent proliferation of generative large language models such as *ChatGPT*. With an increased push to deploy these new technologies in health care, we must have a clear understanding of the issues with health care data as it currently exists. Many participants agreed that in health care applications, close human involvement in the creation and implementation of these tools is vital. Although AI may complement and assist physicians, it cannot currently replace the expertise and contextual understanding of an experienced medical provider. Interdisciplinary collaboration between medical professionals and AI experts will ensure that models are both technically sound and clinically relevant. The interest and engagement in our conference illustrates the need for additional and expanded partnership between data scientists and physicians.

As AI applications become more common in the clinical environment, physician education will need to include content to understand how to safely apply these models to patient care. Many AI technologies are designed for nonmedical applications and contexts. The motivations, incentives, and consequences of error in clinical applications are unique, and the clinical and experiential knowledge of medical providers will be essential in ensuring these models are correctly calibrated and optimized.

Trust and data privacy issues are similarly unique to clinical information. Health care privacy regulations such as HIPAA are particularly strict, posing challenges in accessing and using clinical data. The development of AI solutions must strike a balance between protecting patient privacy and ensuring data accuracy. Building and maintaining trust at both the patient and community levels is essential for the sustainability and success of AI applications in health care. Community engagement, academic accountability, and transparency in research are all key elements in fostering this trust. Our conference did not yield a consensus on solutions to maximize privacy and accuracy, and this is an important area for future research.

The extent of concern regarding missingness in health care data and its potential ramifications on incorporating machine learning into health care was apparent in the considerable interest our conference attracted. With 861 registered participants and 164 attending the live event, many experts in the field are intent on discussing this issue and devising solutions. Engagement with the conference, evidenced by an average attendee score of 8.8, further emphasizes the value of this topic among data scientists and physicians.

### Recommendations

Based on the findings of our conference and analysis, we propose the following recommendations for addressing data quality, human input, and trust challenges in AI applications for health care:

- Data quality and bias
  Expand data collection efforts to reduce gaps and biases in health care data by including diverse patient populations and multiple data sources and types.
  Apply formal statistical methods to evaluate data quality and reliability of AI models and data imputation techniques to account for missing data with awareness of potential biases and inaccuracies introduced.
  Develop new strategies to better identify and address missing data.
- Human input in model development and deployment
  Test and evaluate machine learning models with community engagement for accuracy, fairness, and generalizability.
  Adopt community-based data collection approaches that involve communities as active members in the research process.
  Include medical professionals in the development and implementation of AI models.
- Trust and privacy
  Collect data ethically and fairly, ensuring the inclusion of underrepresented communities and social predictors of health.
  Prioritize communication, transparency, and respect for privacy to cultivate trust with patients and health care providers.
  Develop clear ethical guidelines to safeguard patient privacy, balancing the need for accurate data with the protection of personal information.

### Limitations

We acknowledge several important limitations of our study. The transferability of our data may be limited by subject selection, as the selection of our speakers and topics of discussion may not encompass every aspect of the missingness problem in health care data. Although we endeavored to select speakers with a wide variety of views, it is noteworthy that none of our speakers expressed substantial criticism toward the expansion of data collection in the health care system. The absence of dissenting voices that are present in other fields could be a result of our speaker selection, but we suspect that in health care, the critical perspective toward data collection is less prevalent. This could be attributed to the fact that data collection is fundamentally interwoven with the practice of patient care, rendering the premise of *not* collecting data untenable.

However, we were able to recruit a diverse group of participants with varied backgrounds and collected rich textual data, and our data achieved thematic sufficiency to generate meaningful conclusions in our analysis. Notably, patient perspectives were not included as part of the goals of our conference. Future investigations into patient attitudes and perspectives on AI in health care should be conducted. Additionally, as a single event, conclusions and recommendations should be viewed as an initial step rather than exhaustive solutions. Given the dynamic nature of both health care and AI, these challenges and potential solutions are likely to evolve over time. As such, ongoing discussions and research are needed to ensure these conclusions remain relevant.

The thematic analysis conducted in this study is subject to several limitations. Data were gathered during a live broadcast of our event, so opinions may have been impacted by social desirability bias. It is possible that participant views may differ with a different method of data collection, but we aimed to collect broad perspectives by asking open-ended questions and promoting a diversity of opinions. As this research was conducted in a constructivist paradigm, the research team was actively involved in generating meaning from the data. We used group consensus and multiple independent analyses to minimize individual reflexivity; however, we acknowledge that the research team’s personal experience and background influenced our results. We have attempted to be transparent about our background and the potential influence of our perspectives. Finally, our goal was the description of themes and perspectives, and further work should be conducted to investigate the theoretical underpinning of these perspectives.

### Conclusions

The *Missingness in Action* conference highlighted the importance of addressing missingness in health care data, which directly impacts the successful implementation of machine learning in health care by improving its accuracy and applicability. The conference was well received by its audience and facilitated insightful discussions and proposed promising strategies that emphasize the importance of data quality and bias, human input in model development and deployment, and trust and privacy. The strategies discussed during the conference provide a starting point for addressing this problem, but further research and collaboration is needed to develop and implement effective solutions.

## Abbreviations

AI: artificial intelligence
HAI: Human-Centered Artificial Intelligence
HIPAA: Health Insurance Portability and Accountability Act
